# Gastrointestinal Complaints During Exercise: Prevalence, Etiology, and Nutritional Recommendations

**DOI:** 10.1007/s40279-014-0153-2

**Published:** 2014-05-03

**Authors:** Erick Prado de Oliveira, Roberto Carlos Burini, Asker Jeukendrup

**Affiliations:** 1 School of Medicine, Federal University of Uberlândia, Av. Pará, no 1720 Bloco 2U, Campus Umuarama, Uberlândia, Minas Gerais 38400-902 Brazil; 2 Centre for Physical Exercise and Nutrition Metabolism, UNESP School of Medicine, Public Health Department, Botucatu, São Paulo Brazil; 3 Gatorade Sports Science Institute, Barrington, IL USA; 4 School of Sport, Exercise and Health Sciences, Loughborough University, Loughborough, UK

## Abstract

Gastrointestinal problems are common, especially in endurance athletes, and often impair performance or subsequent recovery. Generally, studies suggest that 30–50 % of athletes experience such complaints. Most gastrointestinal symptoms during exercise are mild and of no risk to health, but hemorrhagic gastritis, hematochezia, and ischemic bowel can present serious medical challenges. Three main causes of gastrointestinal symptoms have been identified, and these are either physiological, mechanical, or nutritional in nature. During intense exercise, and especially when hypohydrated, mesenteric blood flow is reduced; this is believed to be one of the main contributors to the development of gastrointestinal symptoms. Reduced splanchnic perfusion could result in compromised gut permeability in athletes. However, although evidence exists that this might occur, this has not yet been definitively linked to the prevalence of gastrointestinal symptoms. Nutritional training and appropriate nutrition choices can reduce the risk of gastrointestinal discomfort during exercise by ensuring rapid gastric emptying and the absorption of water and nutrients, and by maintaining adequate perfusion of the splanchnic vasculature. A number of nutritional manipulations have been proposed to minimize gastrointestinal symptoms, including the use of multiple transportable carbohydrates, and potentially the use of nutrients that stimulate the production of nitric oxide in the intestine and thereby improve splanchnic perfusion. However, at this stage, evidence for beneficial effects of such interventions is lacking, and more research needs to be conducted to obtain a better understanding of the etiology of the problems and to improve the recommendations to athletes.

## Introduction

Gastrointestinal complaints are very common among endurance athletes. Anecdotally, gastrointestinal problems are perhaps the most common cause of underperformance in endurance events. Depending on the methodology used and the events studied, an estimated 30–90 % of distance runners experience intestinal problems related to exercise [1, 2]. These complaints may be of varying severity, but symptoms may include nausea, vomiting, abdominal angina, and bloody diarrhea. In many cases, these problems have not only negative effects on performance but also an impact on subsequent recovery. The presence and nature of abdominal symptoms experienced by athletes vary from mild, exercise-related discomfort to severe ischemic colitis and diarrhea [3]. Bill Rodgers, marathon legend, with four victories in both the Boston marathon and the New York City marathon in the late 1970s said, “More marathons are won or lost in the porta-toilets than at the dinner table.” This illustrates the magnitude of the problem for endurance athletes, particularly long-distance runners. This review discusses the prevalence of gastrointestinal complaints in athletes and the etiology of the problems, and begins to develop guidelines to prevent the issues.

## Methodological Considerations

Clearly, there is large variation in the reported prevalence in the literature and this seems to be attributable to, among other factors, the method of investigation (and the way in which gastrointestinal symptoms are defined and recorded). However, in addition, the reported prevalence of these symptoms varies in different studies depending on the study population, sex, age, and training status of the athletes, as well as the mode and intensity of the exercise studied, and the environmental conditions. There is also no consistent definition of what constitutes a severe or non-severe symptom. It has been suggested that a severe symptom is any symptom that impacts performance and/or health. So, for example, very mild nausea may not affect performance, but a higher degree of nausea is likely to have negative consequences. Flatulence is unlikely to have effects on performance, but vomiting, independent of the severity score provided by the athlete, is likely to have a negative impact on performance. This is an approach discussed by the authors in a paper in 2000 [2] and it is suggested that future studies in this area use a similar categorization of symptoms and scoring approach.

When analyzing the reported symptoms, it becomes immediately obvious that they are highly individual and there are no clear patterns with regard to the type of activity and the types of symptoms observed. There is a fairly large, yet well defined number of different gastrointestinal symptoms that can occur during exercise. Generally, the symptoms can be classified as either upper or lower gastrointestinal tract. Typically, lower gastrointestinal tract problems are more severe in nature, but all symptoms have the potential to impair performance. Symptoms are often mild and may not affect performance, but they can also be very serious and can not only affect performance, but also be health threatening.

## Prevalence of Gastrointestinal Symptoms in Athletes

Gastrointestinal symptoms are common in many sports but particularly in endurance events. One early review stated that in exhausting endurance events, 30–50 % of participants may experience one or more gastrointestinal symptoms [1]. A study in long-distance triathletes who competed in extreme conditions demonstrated a prevalence of up to 93 % for any gastrointestinal symptom [2]. Among elite endurance athletes, the prevalence of exercise-induced gastrointestinal symptoms was reported to be 70 % [4], and in an internet-based observational study of 1,281 athletes, 45 % reported at least one gastrointestinal symptom [5]. Gastrointestinal distress is a pervasive problem, especially in ultra-endurance events [6–9]. Nausea, vomiting, abdominal cramping, and diarrhea have been reported in 37–89 % of runners participating in races 67–161 km long [6–9], and fecal blood loss indicating gastrointestinal hemorrhage was reported in 85 % of participants in a 161 km ultra-marathon [6]. A recent study investigated gastrointestinal problems in a group of ultra-marathon runners, and observed that 9 of 15 runners experienced gastrointestinal distress, including nausea (89 %), abdominal cramps (44 %), diarrhea (44 %), and vomiting (22 %) [9].

The prevalence of symptoms varies considerably depending on the event, the environmental conditions, and the level of the athlete. Pfeiffer et al. [10] reported severe gastrointestinal distress ranging from 4 % in marathon running and cycling up to 32 % in Ironman races. Large differences were observed not only between the different events, but also between individuals within the same event. It was demonstrated that there was a strong correlation between gastrointestinal complaints and having a history of gastrointestinal symptoms, indicating that some people are more prone to developing gastrointestinal symptoms [10, 11], and suggesting that there is a large genetic component to these problems.

Gastrointestinal symptoms are not only inconvenient, they can also affect performance and, in extreme cases, have longer-term health implications. In one study, 43 % of triathletes reported serious gastrointestinal problems, and 7 % abandoned the race because of gastrointestinal problems [2]. In two 161-km ultra-marathons, nausea and/or vomiting were the main reasons for dropping out among non-finishers and were the second most common problem impacting race performance among finishers [7]. Clearly, gastrointestinal problems can have major effects on performance.

## Gastrointestinal Symptoms and Pathophysiology

Among the reported deleterious manifestations of strenuous exercise are mucosal erosions and ischemic colitis, both observed after long-distance running [12–14]. For example, marathon runners and long-distance triathletes occasionally have blood loss in the feces in the hours following a marathon. Schaub et al. [15] observed epithelial surface changes known to occur during ischemia on colonoscopic inspection of one such triathlete following a marathon, and suggested that ischemia of the lower gastrointestinal tract induced the problems. Blood loss as a result of ischemic colitis is not uncommon in athletes, and can be profound in extreme cases. Proximal, distal, or pancolitis, and even small bowel, infarctions have been reported in athletes and in some cases have required surgery [14, 16]. Despite the high prevalence of mild or severe symptoms, the etiology of these gastrointestinal complaints in endurance athletes is still incompletely understood.

## Causes of Gastrointestinal Problems

While it is recognized that the etiology of exercise-induced gastrointestinal distress is multifactorial, gastrointestinal ischemia is often acknowledged as the main pathophysiological mechanism for the emergence of the symptoms [5, 17, 18]. The other factors are mechanical and nutritional in nature.

### Effects of Exercise on Gut Function

The effects of exercise on gastrointestinal function have been studied in different ways. Traditionally, the research focused on gut perfusion, but more recently the effects of exercise on motility, gut barrier function, and absorption have been studied. Having an understanding of the physiology of the gut during exercise is essential to elucidating the factors that may contribute to the development of gastrointestinal symptoms.

#### Splanchnic Hypoperfusion

Large heterogeneity exists in the response of the gastrointestinal system to exercise. Splanchnic hypoperfusion during exercise ranges from mild circulatory changes to profound gastrointestinal ischemia [19]. In line with this, the consequences of hypoperfusion within the gastrointestinal tract (i.e., epithelial injury and changes in gastrointestinal permeability and epithelial barrier function) differ greatly between individuals. During strenuous physical activity or exercise, norepinephrine is released from nerve endings and on binding to α-adrenoreceptors of the sympathetic nervous system, induces splanchnic vasoconstriction. This will result in an increase in the total splanchnic vascular resistance [20, 21], while, at the same time, vascular resistance with increased activity during exercise in other tissues, such as the heart, lungs, active muscle, and skin, is decreased [22, 23]. During maximal exercise, splanchnic blood flow may be reduced by up to 80 % to provide sufficient blood flow to working muscle and skin. As blood is shunted from viscera to the active tissues [23], gut mucosal ischemia may result, as well as increases in mucosal permeability [19, 24]. This in turn may be linked to nausea, vomiting, abdominal pain, and diarrhea [17, 25], although convincing evidence for this is lacking [26].

#### Changes in Motility

Changes in motility might be observed at different levels of the intestinal tract: the esophagus, the stomach, and the intestine. Decreases in esophageal peristaltic activity, a decrease in lower esophageal sphincter tone, and increased transient lower sphincter relaxation have been observed and could be linked to gastro-esophageal reflux during exercise [27]. The effects on gastric emptying are less clear, but several studies, including a very early study [28], reported no effect of moderate exercise on gastric emptying. However, during exercise at very high intensity or during intermittent activity, gastric emptying may be affected [29].

When gastric emptying was studied using the Loughborough intermittent shuttle test, gastric emptying of fluids was reduced approximately by half. Exercising in the heat per se did not appear to affect gastric emptying very much, except at extreme temperatures (49 °C) [30]. However, exercising in a hypohydrated state does seem to significantly affect gastric emptying [30, 31].

Studies performed so far suggest that the effects of exercise on the small bowel as well as on the colon are limited. In one study, motility was investigated in symptomatic and asymptomatic runners using a telemetric pH sensor [32]. It was observed that small bowel and colonic transit times were similar in the two groups at rest. Interestingly, although diarrhea was reported in that study, there was no link with colonic transit time and therefore there must be another cause for the observed gastrointestinal symptoms.

Overall, it seems that moderate exercise has little effect on gastrointestinal tract motility, but when exercise becomes more severe, there may be some inhibiting effects, especially at the level of gastric emptying.

#### Absorption and Gut Permeability

Studies also suggest that exercise has little effect on intestinal absorption of both water and carbohydrate [33, 34]. However, it must be noted that most studies used exercise intensities that were moderate and durations of exercise that were no longer than 2 h. It is feasible that, during higher intensities of exercise when intestinal blood flow is more compromised, and also after more prolonged exercise, that absorption could be reduced. It has also been reported that with fluid restriction intestinal permeability may be increased [35], possibly because dehydration ultimately influences gut perfusion.

Oktedalen et al. [36] reported increased intestinal permeability after a marathon, indicating damage to the gut and impaired gut function. There are numerous techniques available to study gut permeability but to date there is limited information. The information that is available suggests that gut permeability can be compromised in athletes [24]. Although this has not been conclusively linked to gastrointestinal symptoms, one study showed that gut permeability in symptomatic runners was greater than in asymptomatic runners [37]. On the other hand, in one long-distance triathlon in extreme conditions in which gastrointestinal symptoms were highly prevalent, no compromised gut barrier function was observed as measured by bacterial translocation (lipopolysaccharide stimulation). This technique is a marker of mucosal damage and the invasion of Gram-negative intestinal bacteria and/or their toxic constituents (endotoxins) into the blood circulation [2]. More research needs to be conducted before there is a clear understanding of the causes of gastrointestinal distress.

#### Summary of the Effects of Exercise on Gut Function

In summary, exercise results in numerous changes in the intestinal tract, and most of these effects are intensity dependent. Many of the functions are not affected at low-intensity exercise but become progressively affected at higher intensities. The changes include a reduction in mesenteric blood flow, decreases in esophageal peristaltic activity, decreases in lower esophageal sphincter tone, and increased transient lower sphincter time and a reduction of gastric emptying. As a result of reduced gastrointestinal tract perfusion, absorption might be affected and gut barrier function might be compromised.

### Mechanical Causes

The mechanical causes of gastrointestinal problems are related to either impact or posture. For example, symptoms are more common in runners than in cyclists [27]. This is thought to be a result of the repetitive high-impact mechanics of running and subsequent damage to the intestinal lining [38]. This repetitive gastric jostling is also thought to contribute to lower gastrointestinal symptoms such as flatulence, diarrhea, and urgency. The mechanical trauma suffered by the gut from the repetitive impact of running in combination with gut ischemia is probably the cause of the bleeding [3, 25].

Posture can also have an effect on gastrointestinal symptoms. For example, on a bicycle, upper gastrointestinal symptoms are more prevalent possibly due to increased pressure on the abdomen as a result of the cycling position, particularly when in the ‘aero’ position. ‘Swallowing’ air as a result of increased respiration and drinking from water bottles can result in mild to moderate stomach distress. In general, the only way to reduce the effects of these mechanical causes is by training [3, 39].

### Nutritional Causes

It is known that nutrition can have a strong influence on gastrointestinal distress, although many of the problems can occur in the absence of any food intake before or during exercise. Fiber, fat, protein, and fructose have all been associated with a greater risk of developing gastrointestinal symptoms. Dehydration, possibly as a result of inadequate fluid intake, may also exacerbate the symptoms. A study by Rehrer et al. [40] demonstrated a link between nutritional practices and gastrointestinal complaints during a half-Ironman triathlon. Gastrointestinal problems were more likely to occur with the ingestion of fiber, fat, protein, and concentrated carbohydrate solutions during the triathlon. Beverages with high osmolalities (>500 mOsm/L) seemed to be associated with an increased incidence of symptoms.

It appears that foods that delay gastric emptying and might cause a shift of fluids into the intestinal lumen are more likely to cause gastrointestinal symptoms. Highly concentrated carbohydrate solutions with high osmolalities could have this effect. Although subject numbers were too small to perform meaningful statistical analysis, a study by Wallis et al. [41] reported more severe gastrointestinal symptoms in women with a high carbohydrate intake (1.0 or 1.5 g/min) than in those with a low intake (0 or 0.5 g/min). However, the data are equivocal. For example, in a field study in which runners ran two 16-mile races while consuming carbohydrate at a high rate (1.4 g/min) in the form of gels, very few gastrointestinal symptoms were observed (~10 % of runners reported symptoms) [11]. Perhaps the duration of the exercise was not long enough to cause significant gastrointestinal distress, or, alternatively, carbohydrate content per se is not as important a factor as has often been assumed. In a larger study, with 221 endurance athletes competing in various events including marathons and Ironman races, carbohydrate intake was positively correlated to nausea and flatulence [10]. However, nausea was relatively mild and a higher carbohydrate intake was also correlated with faster finish times, suggesting that nausea did not have any negative effects on performance. Of course, no causal relationships can be obtained from these correlations so the data must be interpreted with caution.

It may be speculated that it is not simply carbohydrate intake that causes gastrointestinal symptoms, but it may be a complex interplay of a number of factors such as carbohydrate concentration, the type of carbohydrate, osmolality and acidity of a beverage that might be linked with gastrointestinal problems. More research is definitely needed to find carbohydrate solutions that reduce the risk of developing gastrointestinal symptoms.

## Nutritional Solutions to Gastrointestinal Problems

Although much is still unknown about the etiology of gastrointestinal symptoms, a number of nutritional manipulations have been suggested to reduce the number or the severity of symptoms. For athletes who compete in endurance events, an interesting observation is that when a beverage is consumed that contains multiple transportable carbohydrates such as glucose and fructose, gastrointestinal symptoms seem to be reduced compared with the consumption of the same (large) amount of a single carbohydrate (glucose). This has been a consistent finding in a number of studies from the authors’ laboratory [42–45]. Rowlands et al. [46] recently reported fewer gastrointestinal problems with multiple transportable carbohydrates (maltodextrin : fructose) in mountain bikers, and concluded that the ingestion of multiple transportable compares with a single carbohydrate-enhanced mountain bike race and high-intensity laboratory cycling performance. These results were found with inconsistent, but not irreconcilable, effects of gut discomfort as a possible mediating mechanism.

More recently, van Wijck et al. [19] argued that, if perfusion of the gut is one of the main causes of the problems, then manipulation of blood flow to the gut by upregulating nitric oxide production could be a way to reduce symptoms. There are a number of options to upregulate intestinal nitric oxide availability that may be of value for athletes experiencing abdominal distress associated with splanchnic hypoperfusion, including nitric oxide synthase-dependent (glutamine–arginine–citrulline) and nitric oxide synthase-independent (nitrate–nitrite) supplementation. Research is in its infancy, but of the ingredients that have been studied, dietary nitrate probably has the most potential to influence splanchnic perfusion [19]. However, current recognized vegetable sources of dietary nitrate are also sometimes associated with gastrointestinal symptoms.

## ‘Training the Gut’

For many years, it has been suggested that the gut can be ‘conditioned’ or ‘trained’ in order to reduce gastrointestinal distress, yet research in this area is still in its infancy [47, 48]. It has been shown that athletes who are not accustomed to fluid and food ingestion during exercise had a twofold risk of developing gastrointestinal symptoms than athletes who were accustomed to taking fluid and food during exercise [5]. In a study by Cox et al. [49], the adaptability of the gut was nicely demonstrated. In that study, 16 endurance-trained cyclists or triathletes were pair matched and randomly allocated to either a high carbohydrate group (high group; *n* = 8) or an energy-matched low carbohydrate group (low group; *n* = 8) for 28 days. It became apparent after 28 days that the high carbohydrate group had higher exogenous carbohydrate oxidation rates during exercise [49]. The higher rates were attributed to improved absorption, which is generally associated with improved tolerance of fluids and foods during exercise, and thus the training would have reduced the chances of gastrointestinal distress [48].

However, it also appears that training with a nutrition strategy can improve tolerance and stomach comfort, independent of changes in adaptations in gut function. Lambert et al. [50] assessed tolerance to fluid ingestion with repeated sessions of drinking while running. During five runs, subjects drank a volume of the solution every 10 min equal to their sweat production over 10 min. Although repeated sessions of drinking at a rate matching sweat rate improved stomach comfort, the gastric emptying rate did not change under such conditions.

More research is needed to understand the adaptations that occur in the intestinal tract, as well as the time course of such changes. This is essential to develop guidance for athletes on how often certain nutritional strategies need to be practised and the best way to conduct such training sessions.

## Non-Steroidal Anti-Inflammatory Drugs

Large numbers of athletes have reported using analgesics to relieve existing or anticipated pain [51]. The use of non-selective non-steroidal anti-inflammatory drugs (NSAIDs) has been associated with a three- to fivefold increased risk of upper gastrointestinal complications, mucosal bleeding, or perforation compared with no medication [52]. In a study at the Chicago marathon [53], it was found that ibuprofen (but not aspirin) ingestion during prolonged exercise may have increased gastrointestinal permeability and led to gastrointestinal symptoms. van Wijck et al. [54] recently demonstrated that ibuprofen aggravates exercise-induced small intestinal injury and induces gut barrier dysfunction, and concluded that the consumption of NSAIDs by athletes is not harmless and should be discouraged in those who experience persistent or recurring gastrointestinal symptoms.

## Practical Implications and Advice to Athletes

In order to prevent gastrointestinal distress, a few guidelines can be provided. However, it must be noted that these are based on limited research. Nevertheless, anecdotally, these guidelines seem to be effective:

- Avoid high-fiber foods in the day or even days before competition. For the athlete in training, a diet with adequate fiber will help keep the bowel regular.
- Avoid aspirin and NSAIDs such as ibuprofen. Both aspirin and NSAIDs have commonly been shown to increase intestinal permeability and may increase the incidence of gastrointestinal complaints. The use of NSAIDs in the pre-race period should be discouraged, mainly for athletes with a history of gastrointestinal problems.
- Avoid high-fructose foods (in particular drinks that are exclusively fructose). However, interestingly, a fructose and glucose combination may not cause problems and may be better tolerated.
- Avoid dehydration. As dehydration can exacerbate gastrointestinal symptoms, it is important to prevent dehydration. Start the race (or training) well hydrated.
- Ingest carbohydrates with sufficient water or choose drinks with lower carbohydrate concentrations to prevent very high concentrations and osmolalities in the stomach.
- Practise new nutrition strategies. Make sure to experiment with the pre-race and race-day nutrition plan many times before the race day. This will allow the athlete to work out what does and does not work, and reduces the chances of getting gastrointestinal symptoms.

## Conclusions

The gut is an important athletic organ because it is responsible for the delivery of water and nutrients during exercise. Both upper and lower gastrointestinal complaints are highly prevalent among athletes during exercise (especially endurance athletes), and can negatively impact performance. In severe cases, health risks can also be present. Most gastrointestinal complaints during exercise are mild and of no risk to health, but hemorrhagic gastritis, hematochezia, and ischemic bowel can present serious medical challenges. Nutritional training and appropriate nutritional choices (avoiding protein, fat, fiber, and milk products) can reduce the risk of gastrointestinal discomfort during exercise by ensuring rapid gastric emptying and the absorption of water and nutrients and by maintaining adequate perfusion of the splanchnic vasculature.
